# A Chinese herbal decoction, reformulated from Kai-Xin-San, relieves the depression-like symptoms in stressed rats and induces neurogenesis in cultured neurons

**DOI:** 10.1038/srep30014

**Published:** 2016-07-22

**Authors:** Lu Yan, Qinghua Hu, Marvin S. H. Mak, Jianshu Lou, Sherry L. Xu, Cathy W. C. Bi, Yue Zhu, Huaiyou Wang, Tina T. X. Dong, Karl W. K. Tsim

**Affiliations:** 1 Division of Life Science, Center for Chinese Medicine, The Hong Kong University of Science and Technology, Clear Water Bay, Hong Kong, China; 2 Shenzhen Research Institute, The Hong Kong University of Science and Technology, Shenzhen, 518057, China; 3 College of Pharmacy, China Pharmaceutical University, Nanjing, China; 4 Jiangsu Collaborative Innovation Center of Chinese Medicinal Resources Industrialization, Nanjing University of Chinese Medicine, Nanjing, China

## Abstract

Kai-Xin-San (KXS), a Chinese herbal decoction for anti-depression, is a combination of paired-herbs, i.e. Ginseng Radix et Rhizoma (GR)-Polygalae Radix (PR) and Acori Tatarinowii Rhizoma (ATR)-Poria (PO). The make-up of the paired-herbs has been commonly revised according to syndrome differentiation and treatment variation of individual. Currently, an optimized KXS (KXS_2012_) was prepared by functional screening different combination of GR-PR and ATR-PO. The aim of this study was to verify the effect and underlying mechanism of KXS_2012_ against depression in chronic mild stress (CMS)-induced depressive rats and in primary cultures of neurons and astrocytes. In rat model, the CMS-induced depressive symptoms were markedly alleviated by the treatment with KXS_2012_. The CMS-suppressed neurotransmitter amounts were restored in the presence of KXS_2012_. And the expressions of neurotropic factors and its corresponding receptors were increased under KXS_2012_ administration. In cultured neurons, application of KXS_2012_ could promote neurogenesis by inducing the expression of synaptotagmin and dendritic spine density. Moreover, application of KXS_2012_ in cultured astrocytes, or in H_2_O_2_-stressed astrocytes, induced the expressions of neurotrophic factors: the increase might be associated with the modification of Erk1/2 and CREB phosphorylation. Our current results fully support the therapeutic efficacy of KXS_2012_ against depression in cell and animal models.

Depression, also known as major depressive disorder, is a serious mental illness characterized by constant feeling of low self-esteem and loss of interest or pleasure^1^. Depression is a complex disease with multiple genetic and environmental pathogenesis. The main pathological changes of depression include neuron/synapse reduction, change in neurotransmitter system and loss of neurotrophic factors, especially in cortex and hippocampus^2^. In addition to neurodegenerative symptoms, untreated depression may progress to suicide or metabolic disorders (diabetes and cardiovascular diseases)^3^,^4^. However, current anti-depression drugs that possess severe side effects are not effective to all the patients. Thus, developing anti-depression drug is urgently needed^5^.

Kai-Xin-San (KXS), a traditional Chinese medicine (TCM), was firstly recorded in *Beiji Qianjin Yaofang* < *Thousand Formulae for Emergency* > by Sun Simiao (581–685 A.D.) of Tang Dynasty in 652 A.D^6^,^7^. KXS has been used in treating mental disorders, especially depression for thousands of years in China. In animal studies, KXS was shown to relieve depression-like symptoms^8^,^9^,^10^, and the mechanism was proposed to be mediated by increasing the amounts of neurotransmitters and neurotrophic factors in the brain^11^. In cell cultures, the functions of KXS in regulating the expressions of neurotrophic factors in cultured astrocytes and neurons were reported^12^,^13^.

KXS composes of two paired-herbs, i.e. Ginseng Radix et Rhizoma (GR; root and rhizome of *Panax ginseng* C. A. Mey.; Araliaceae family) - Polygalae Radix (PR; root of *Polygala tenuifolia* Wild.; Polygalaceae family) and Acori Tatarinowii Rhizoma (ATR; rhizome of *Acorus tatarinowii* Schott; Acoraceae family) - Poria (PO; sclerotium of *Poria cocos* (Schw.) Wolf; Polyporaceae family). The make-up of these two paired-herbs in KXS has been commonly changed according to syndrome differentiation and treatment variation, which causes confusion in clinics. Thus, an optimized KXS (KXS_2012_, GR-PR: ATR-PO = 1:5) was prepared by functional screening the combination of GR-PR and ATR-PO^14^. Here, we propose it is reasonable to use KXS_2012_ to attenuate depression. With this in mind, we evaluate the functions of KXS_2012_ against depression on cell and animal studies.

Among different depressive animal models, the chronic mild stress (CMS)-induced depressive rat is usually selected for its similarity with the true state of depressive patients^15^,^16^. Thus, the CMS-induced depressive rat model promises a stable and credible approach in evaluating the function of anti-depressants, and the study on tissue samples from depressive rats indicates the underlying mechanism against depression. Moreover, neuron/synapse reduction is one of the most severe pathogenesis during depression. In depressed brain, nerve reduction supports the neurogenesis theory of depression, which posits that depression is largely caused by an impairment of the brain’s ability to produce neurons, a process known as neurogenesis. This disorder is reversible when depressed patients are treated, which presents a novel and effective therapeutic target against depression^17^,^18^. The study on neurogenesis using neuron and astrocyte cultures predicts the potential cellular mechanism against depression, which helps to represent biological functionality and integration of molecular information. In this study, we aimed to discover the functions of KXS_2012_ against depression as well as the potential underlying mechanism. Our results could accelerate the development of new therapy for anti-depression.

## Results

### Standardization of herbal extracts

GR-PR and ATR-PO extracts were prepared according to ancient preparation of Chinese herbal mixture. The extraction efficiency was about 25.79 ± 3.25% for GR-PR extract and 10.32 ± 2.78% for ATR-PO extract (Mean ± SD, *n* = 3). Two approaches were chosen to control the quality of paired-herb extracts: (i) chemical fingerprinting; and (ii) assessment of chemical markers. Known chemicals were identified in chemical fingerprints (Fig. S1A), which accorded to our published reports^7^,^14^. Eight chemical markers were selected: e.g. GR-derived ginsenosides (Rb_1,_ Rd, Re, Rg_1_), PO-derived pachymic acid, PR-derived 3,6′-disinapoyl sucrose (330 nm) and ATR-derived α-asarone (258 nm) and β-asarone (258 nm) (Fig. S1). In GR-PR extract, the amount in mg/g of dried herbal extract was about 4.287 ± 0.065 for ginsenosides Rb_1_, 0.397 ± 0.046 for ginsenosides Rd, 3.648 ± 0.079 for ginsenosides Re, 3.304 ± 0.084 for Rg_1_ and 9.678 ± 0.067 for 3,6′-disinapoyl sucrose. In ATR-PO extract, the amount in mg/g of dried herbal extract was about 6.292 ± 0.017 for α-asarone, 0.736 ± 0.014 for β-asarone and 0.021 ± 0.003 for pachymic acid (Mean ± SD, *n* = 3). The established chemical parameters served as the control for repeatability of the below animal and biochemical analyses. Thus, KXS_2012_ was referring to a mixture of GR-PR extract plus ATR-PO extract at 1: 5 weight ratio.

### KXS_2012_ reverses the depression-like symptoms in CMS-induced depressive rats

**Figure 1:**
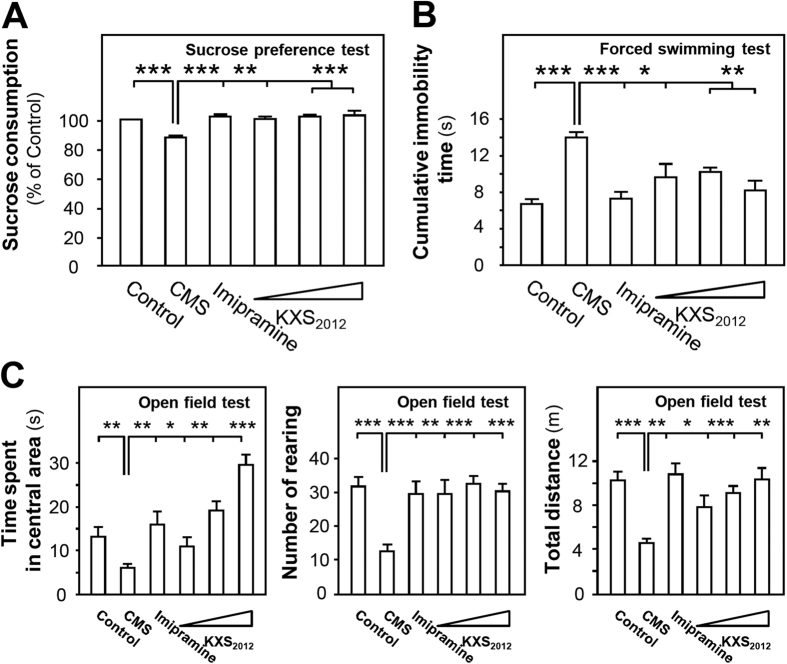
KXS_2012_ alleviates the depression-like symptoms in CMS-induced depressive rats. The CMS-induced depressive rats were randomly divided into six groups: control, CMS model, imipramine (20 mg/kg/day), KXS_2012_ low dose (60.9 mg/kg/day), KXS_2012_ medium dose (182.7 mg/kg/day) and KXS_2012_ high dose (548.1 mg/kg/day). After drug treatment, sucrose preference **(A)**, forced swimming **(B)** and open field tests **(C)** were carried out, as described in the method session. Values are expressed in percentage of un-stressed control, second (s), number or miter (m), Mean ± SEM, *n* = 8, *p < 0.05, **p < 0.01 and ***p < 0.001 compared to the control.

Three animal behavior tests including sucrose preference, forced swimming and open field tests were employed to evaluate the function of KXS_2012_ against depression in rat. After the treatment of herbal extracts for 6 weeks, KXS_2012_ at three doses (low dose: 60.9 mg/kg/day, medium dose: 182.7`mg/kg/day and high dose: 548.1 mg/kg/day) alleviated sucrose preference (~20%) of CMS-induced depressive rats (Fig. 1A). In forced swimming test, the CMS-induced depressive rats doubled cumulative immobility time, while KXS_2012_ at all doses restored the cumulative immobility time (Fig. 1B). In open field test, the CMS-induced depressive rats showed a decrease of over 50% movements. However, the treatment of KXS_2012_ at all doses reversed the time spent in central area of CMS-induced depressive rats: this treatment also affected the horizontal and vertical movements of KXS_2012_-treated rats (Fig. 1C). Imipramine (20 mg/kg/day) was set as a positive control, and non-stressed rats were set as another control.

### KXS_2012_ restores the levels of neurotransmitters in CMS-induced depressive rat brain

According to previous study, a systematic method was used to determine the amounts of neurotransmitters in rat brain by HPLC-fluorescence detector (FLD)^19^,^20^. The detected neurotransmitters included: norepinephrine, dopamine and serotonin. In addition, 5-hydroxyindoleacetic acid (5-HIAA), a major metabolite of serotonin (5-HT), was also determined. The ratio of 5-HIAA/5-HT represents the metabolic state of serotonin. Thus, the ratio of 5-HIAA/5-HT was calculated to interpret the metabolism of serotonin. The HPLC conditions for neurotransmitter detection were determined. The analytic parameters are listed in Table S1. The chromatograms of neurotransmitters are shown in Fig. S2.

**Figure 2:**
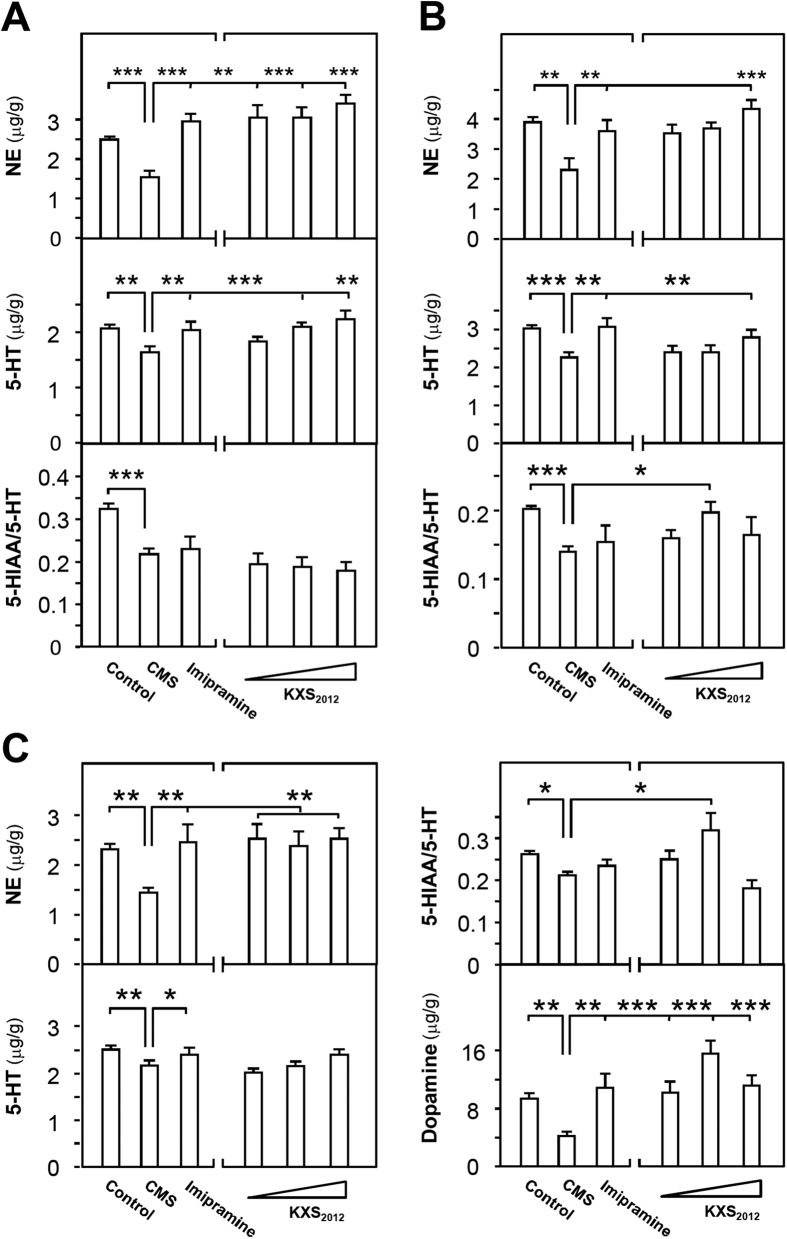
KXS_2012_ restores the levels of neurotransmitters in CMS-induced depressive rat brain. **(A)** The CMS-induced depressive rats were randomly divided into six groups as the protocol in Fig. 1. After drug treatment, the hippocampus was collected. The amounts of norepinephrine (NE), serotonin (5-HT) and ratio of 5-HIAA/5-HT in the extracts of hippocampus were calculated, as described in method session. Similar procedures were applied onto cerebral cortex **(B)** and striatum **(C)** of CMS-induced depressive rats. Additionally, the level of dopamine in striatum **(C)** was determined (bottom right). Dopamine level was too low to be measured in hippocampus and cortex. Values are expressed in μg/g or the number of ratio, Mean ± SEM, *n* = 8, *p < 0.05, **p < 0.01 and ***p < 0.001 compared to the corresponding control.

Three brain regions were selected here, i.e. hippocampus, cerebral cortex and striatum. In CMS-induced depressive rat hippocampus, the amounts of serotonin and norepinephrine, as well as the ratios of 5-HIAA/5-HT, were decreased to ~80%, ~60% and ~65%, respectively (Fig. 2A). The treatment of KXS_2012_ at all doses restored the levels of serotonin and norepinephrine. However, the KXS_2012_ treatment failed to reverse the ratio of 5-HIAA/5-HT caused by CMS (Fig. 2A). In depressive rat cerebral cortex, the reduction in amounts of serotonin (~25%), norepinephrine (~40%) and ratio of 5-HIAA/5-HT (~25%) were observed. KXS_2012_ at all doses restored the levels of serotonin and norepinephrine in cortex. In parallel, KXS_2012_ at medium dose (182.7 mg/kg/day) increased the ratio of 5-HIAA/5-HT to normal state (Fig. 2B). In depressive rat striatum, the amounts of serotonin, dopamine and norepinephrine, as well as the ratio of 5-HIAA/5-HT, were reduced from 20% to 50%. KXS_2012_ at all doses restored the levels of serotonin, dopamine and norepinephrine, as well as the ratio of 5-HIAA/5-HT in the striatum (Fig. 2C). Imipramine (20 mg/kg/day) was used as a positive control, and non-stressed rats were set as another control.

### KXS_2012_ up-regulates neurotrophic factor expressions in CMS-induced depressive rat brain

**Figure 3:**
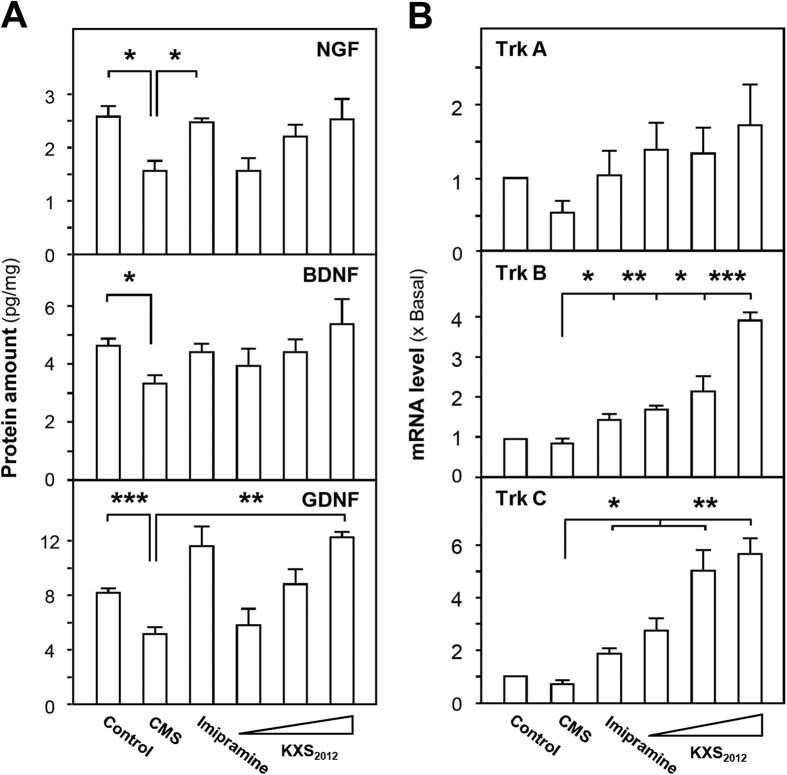
KXS_2012_ restores the levels of neurotrophic factors and their receptors in CMS-induced depressive rat brain. (**A**) The CMS-induced depressive rats were randomly divided into six groups as the protocol as in Fig. 1. After drug treatment, the protein levels of neurotrophic factors (NGF, BDNF and GDNF) in depressive rat cerebral cortex were analyzed by ELISA. The calibration curves of protein amounts were from Fig. S3. **(B)** The mRNA expression levels of neurotrophic factor receptors (Trk A, Trk B and Trk C) in depressive rat cerebral cortex were analyzed by real-time quantitative PCR. Values are expressed in pg/mg of protein or the number of fold to control (x Basal), Mean ± SEM, *n* = 4, *p < 0.05, **p < 0.01 and ***p < 0.001 compared to the corresponding control.

The deficiency of neurotrophic factors is one of the indicative pathogens during depression. Here, the protein levels of nerve growth factor (NGF), brain-derived neurotrophic factor (BDNF) and glia cell-derived neurotrophic factor (GDNF) in depressive rat cerebral cortex were detected by ELISA. The standard curves for calibration were: Y = 0.002X + 0.026 (NGF), *R*^2^ = 0.998; Y = 0.001X−0.044 (BDNF), *R*^2^ = 0.990; Y = 0.000516X + 0.00648 (GDNF), *R*^2^ = 1.000 (Fig. S3). The protein levels of NGF, BDNF and GDNF in cortex were markedly decreased in CMS-induced depressive rat cortex; however, this reduction was alleviated after KXS_2012_ treatment (Fig. 3A). In the same rat samples, the CMS-reduced mRNA expressions of neurotrophic factor receptors, including tropomyosin receptor kinase (Trk) A, Trk B and Trk C, were restored by the treatment of KXS_2012_ (Fig. 3B). In particularly, KXS_2012_ at medium and high doses largely increased the mRNA expression levels of Trk B and Trk C, indicating that KXS_2012_ might exert anti-depression through up-regulating neurotrophic factor signaling pathway (Fig. 3B). The induction of Trk receptors by KXS_2012_ was robust from 2 to 6 folds as compared to the control, which was much better than the positive control imipramine at 20 mg/kg/day.

### KXS_2012_ promotes neurogenesis in cultured neurons

**Figure 4:**
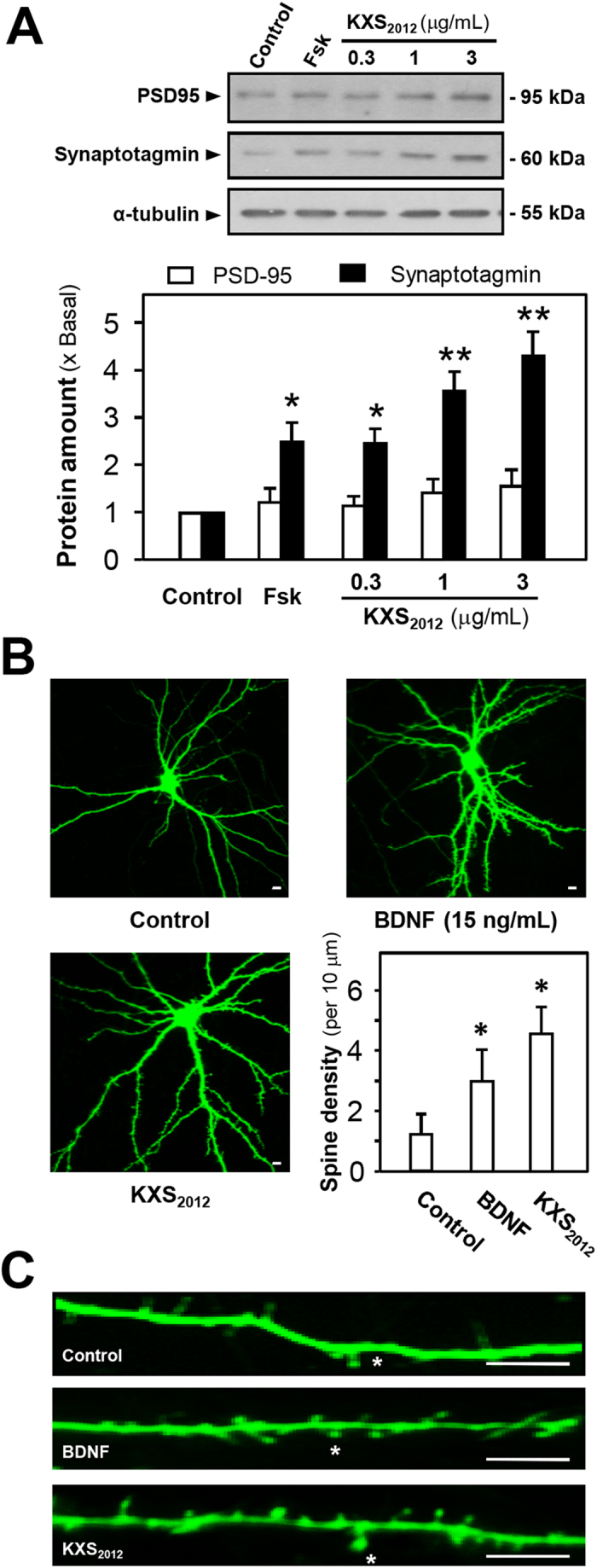
KXS_2012_ promotes neurogenesis in cultured neurons. (**A**) KXS_2012_ (0.3 to 3 μg/mL) was applied onto cultured rat cortical neurons (DIV 5) for 96 h. The protein levels of PSD-95 (~95 kDa) and synaptotagmin (~60 kDa) were measured by Western blot assay (upper panel). α-Tubulin (~55 kDa) served as a loading control. Forskolin (Fsk, 50 nM) was used as a positive control. The amount of protein level was quantified (lower panel). **(B**) KXS_2012_ (3 μg/mL) and BDNF (15 ng/mL) were applied onto cultured hippocampal neurons (DIV 15) for 96 h before pEGFP-N1 transfection. The treatment significantly increased the spine density of control neurons (9–16 dendrites from 6–10 neurons per 10 μm were measured for each condition). The quantification plot was shown in lower right. **(C)** Representative images show that KXS_2012_ (3 μg/mL) and BDNF (15 ng/mL) increased the spine density compared to control. The counted spine density was indicated by asterisk. Scale bars = 10 μm. Values are expressed the fold of change as compared to control (x Basal) or number, Mean ± SEM, *n* = 4–10. *p < 0.05 and **p < 0.01 compared to the control.

The impairment of the brain’s ability to promote neurogenesis underlies depression. As expected, the cellular study of KXS_2012_ on primary cultured rat cortical neurons and hippocampal neurons were employed to investigate the function of KXS_2012_ on neurogenesis. Cultured cortical neurons could undergo the progress of differentiation from day *in vitro* (DIV) 1 to DIV 30. Thus, DIV 5 and DIV 15 were chosen as early and middle stage of neuron differentiation. Synapse formation involves pairing of the pre- and post-synaptic partners at a specific neuro-spatial coordinate^21^,^22^,^23^. The optimized dose of KXS_2012_ in cultured cortical neurons was determined by MTT assays, i.e. the dose did not affect the cell number, as shown in Fig. S4A. In DIV 5 of cortical neurons, KXS_2012_ increased the expression of synaptic vesicle protein, synaptotagmin: the maximal induction could reach over 4 folds (Fig. 4A). Synaptotagmin is a Ca^2+^ sensor regulating neurotransmitter release and hormone secretion. The expression of PSD95, a post-synaptic marker, however was not altered. In DIV 15 of hippocampal neurons, KXS_2012_ increased the dendritic spine density (number of spine per 10 μm segment) at post-synaptic sites by ~4 folds, which was higher than the positive control BDNF application (Fig. 4B,C). The KXS_2012_-induced dendritic spine density could serve as an indicator of increased possible contacts between neurons. In addition, a remarkable increase of synaptotagmin expression was revealed indicating the potential function of KXS_2012_ in synaptogenesis. Thus, the function of KXS_2012_ against depression might be mediated by promoting neurogenesis in the brain.

### KXS_2012_ up-regulates the expressions of neurotrophic factors in normal and H_2_O_2_-stressed astrocytes

**Figure 5:**
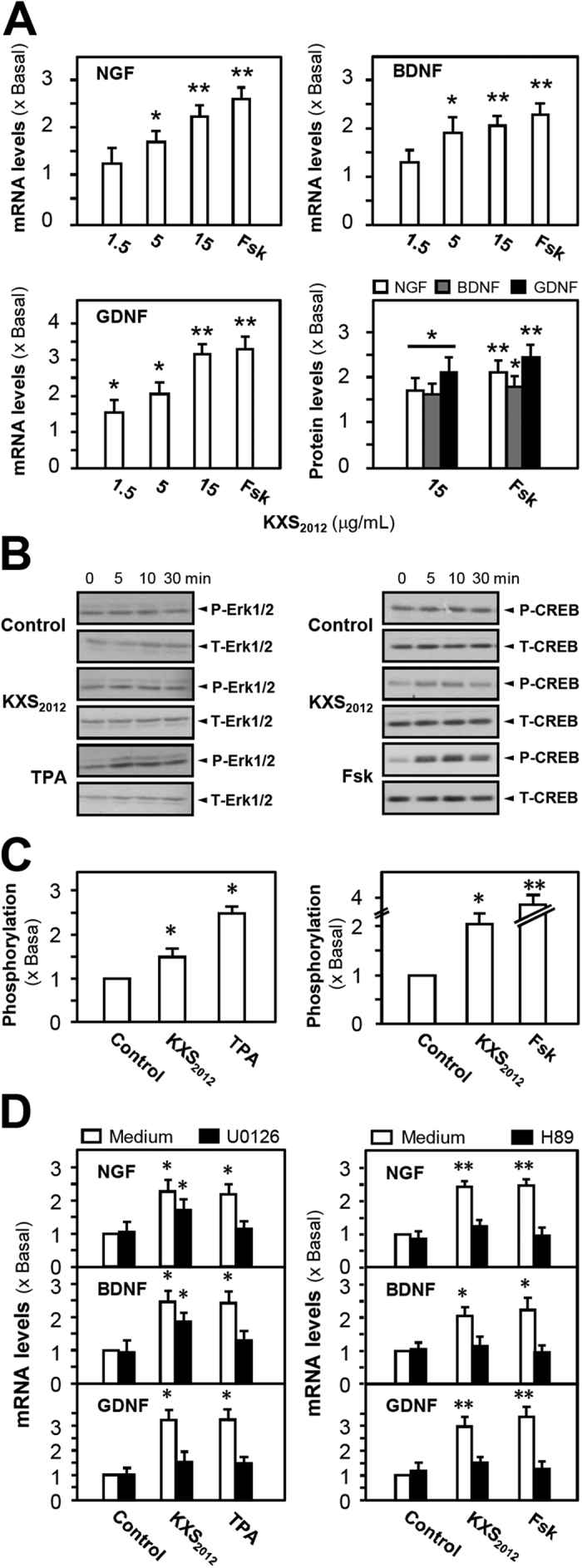
KXS_2012_ up-regulates neurotrophic factor expressions in cultured astrocytes. (**A**) The mRNA and protein levels of neurotrophic factors in cultured astrocytes were analyzed by real-time quantitative PCR and ELISA kits (calibration curves of protein amounts were in Fig. S3). The cultured astrocytes were treated with KXS_2012_ (1.5 to15 μg/mL) for 48 h. Forskolin (Fsk, 50 nM) was used as a positive control. (**B**) Cultured astrocytes were serum starved for 5 h. Then KXS_2012_ (15 μg/mL) was applied onto the cultures. Fsk (50 nM) or TPA (50 nM) served as a positive control, respectively. Total Erk1/2, phosphorylated Erk1/2 (both at ~42/44 kDa), total CREB and phosphorylated CREB (both at ~40 kDa) were revealed by using specific antibodies at different time. (**C**) Quantification plot of the phosphorylation level at 10 min was shown. (**D**) U0126 (20 μM) and H89 (5 μM) were applied onto astrocytes 3 h before the drug treatment. Quantification plot of mRNA and protein levels of neurotrophic factors was shown. Fsk (50 nM) or TPA (50 nM) served as a positive control, respectively. Values are expressed as the fold of change as compared to control (x Basal), where control value is set as 1, Mean ± SEM, *n* = 4, *p < 0.05 and **p < 0.01 compared to the control.

Neurotrophic factors secreted from astrocytes play key roles in neurogenesis, and which are down-regulated in brain during depression. The optimized dose of KXS_2012_ in cultured astrocytes was determined by MTT assays, i.e. the dose did not affect the cell number, as shown in Fig. S4B. In cultured astrocytes, application of KXS_2012_ greatly increased the mRNA and protein levels of NGF, BDNF and GDNF: the maximal induction was at ~2 folds (Fig. 5A). For the underlying mechanism, the expressions of neurotrophic factors are mediated by cAMP-protein kinase A (PKA) and mitogen-activated protein kinases (MAPK) signaling pathways in astrocytes^24^,^25^,^26^. The phosphorylation levels of cAMP response element-binding protein (CREB) and extracellular regulated protein kinases (Erk) 1/2 were determined. In cultured astrocytes, KXS_2012_ increased the phosphorylation of Erk1/2 (~1.5 folds) and CREB (~2 folds) in a time-dependent manner (Fig. 5B,C). Then, H89 and U0126, the inhibitors of PKA and MEK1/2, were selected for further study. In all scenarios, application of both inhibitors in cultured astrocytes blocked the KXS_2012_-induced mRNA levels of NGF, BDNF and GDNF (Fig. 5D). The KXS_2012_-mediated phosphorylation of CREB was fully blocked by H89, and reduced by ~60% with U0126. On the other hand, U0126 fully blocked KXS_2012_-mediated phosphorylation of Erk1/2, while H89 did not show any effect (Fig. S5). These results of inhibitors therefore suggested the activation of KXS_2012_ should be firstly at PKA, then to Erk1/2. Erk1/2, which was reported to be located downstream of PKA, participated in the PKA-mediated CREB phosphorylation^27^. In addition, the condition medium derived from KXS_2012_-treated astrocytes did not significantly increase the expressions of synaptotagmin and PSD-95 in cultured cortical neurons (Fig. S6). These results suggested that the potential functional relationship of KXS_2012_ between neurons and astrocytes might be complicated and need further study.

**Figure 6:**
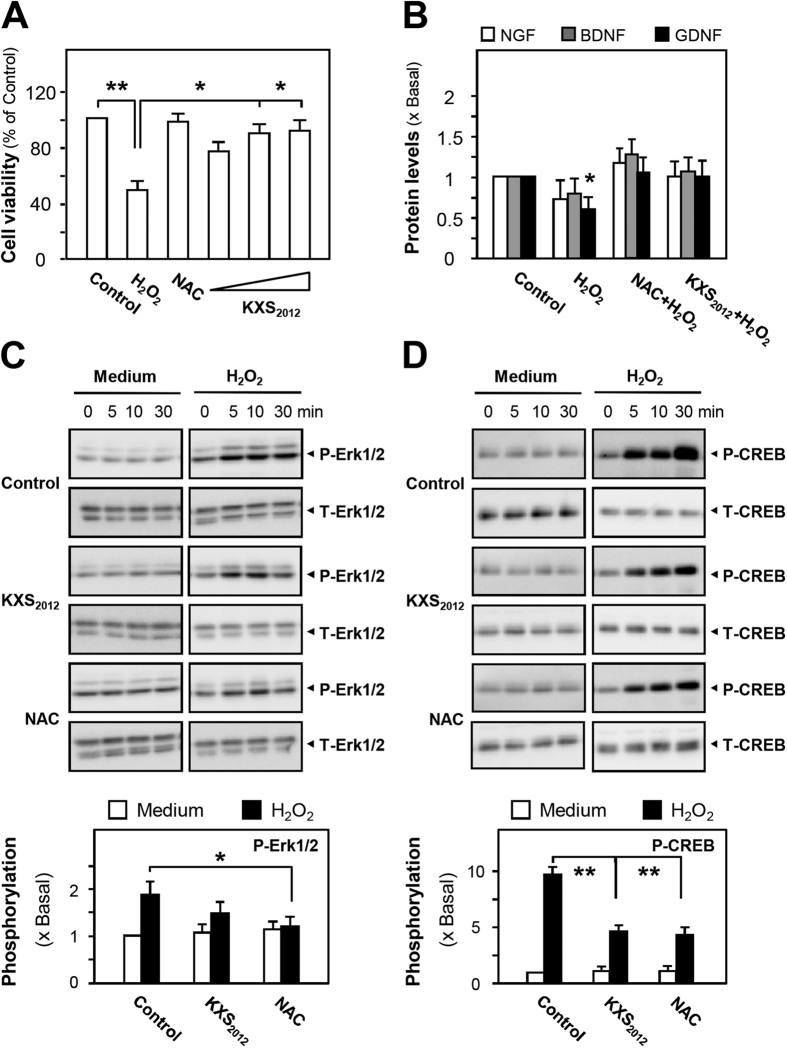
KXS_2012_ up-regulates neurotrophic factor expressions in H_2_O_2_-stressed astrocytes. **(A)** Cultured astrocytes were pre-treated with KXS_2012_ (1.5–15 μg/mL) for 24 h before the addition of H_2_O_2_ (400 μM) for 24 h. The protective effect of herbal extracts by cell viability assay was shown. N-acetyl-L-cysteine (NAC, 1 mM) served as a positive control. The viability of astrocytes is in percentage to normal control. **(B)** Cultured astrocytes were pre-treated with KXS_2012_ (15 μg/mL) for 24 h before the addition of H_2_O_2_ (400 μM) for 24 h. The protein levels of NGF, BDNF and GDNF were determined using ELISA kits (calibration curves of protein amounts were in Fig. S3). NAC (1 mM) served as a positive control. **(C)** Cultured astrocytes were pre-treated with KXS_2012_ (15 μg/mL) for 24 h before the addition of H_2_O_2_ (400 μM). NAC (1 mM) served as a positive control. Total Erk1/2, phosphorylated Erk1/2 (both at ~42/44 kDa) were revealed by using specific antibodies at different time. Quantification plot of the phosphorylation level at 10 min was shown in lower panel. **(D)** Similar procedures were carried out, as described in **(C).** Total CREB and phosphorylated CREB (both at ~40 kDa) were revealed by using specific antibodies at different time. Quantification plot of the phosphorylation level at 30 min was shown in lower panel. Values are expressed as the fold of change as compared to control (x Basal), where control value is set as 1, Mean ± SEM, *n* = 4, *p < 0.05 and **p < 0.01 compared to the control.

Oxidative stress is considered as a common mechanism associated with most neurodegenerative diseases, e.g. depression. Thus, the protective role of KXS_2012_ against oxidative stress was further demonstrated in cultured astrocytes. In H_2_O_2_-stressed astrocytes, the H_2_O_2_-induced toxicity was alleviated by the pre-treatment with KXS_2012_ (Fig. 6A). The H_2_O_2_-induced loss of NGF, BDNF and GDNF proteins were reversed in the presence of KXS_2012_ (Fig. 6B). N-acetyl-L-cysteine (NAC) served as a positive control here. Moreover, the application of KXS_2012_ reduced H_2_O_2_-induced phosphorylations of CREB and Erk1/2 in cultured astrocytes (Fig. 6C,D), which might be associated with the function of neuronal adaptation based on changes in gene expression; these changes of gene expression were shown to be linked to stress^28^,^29^. The differential gene expression related to stress, e.g. induction of apoptotic protein, subsequently down-regulates the expressions of neurotrophic factors. These findings demonstrated that in stressed astrocytes, KXS_2012_ could effectively prevent the loss of neurotrophic factors so as to support neurogenesis, which showed similarity to that of KXS_2012_ in depressive rats.

## Discussion

Depression is a serious mental illness with high incidence in clinics. It causes mood disorder, cognitive and behavioral disturbances, and even increases suicidal tendency^1^. Current treatment of depression is hindered due to the lack of effective agents. Chinese medicine has been widely used in cases of shock, low mood, forbid forgetfulness, anhedonia and dizziness that share similar syndromes to depression. In TCM, depression is manifested as mental disease caused by insufficiency of “xin-qi” and exuberance of “dampness”. KXS is one of the common Chinese herbal formulae for the treatment of depression. In KXS, the two paired-herbs (GR-PR and ATR-PO) with the function of enhancing “xin-qi” and eliminating “dampness” could promise superior efficacy against depression. However, the make-up of these two paired-herbs has been commonly revised according to syndrome differentiation and treatment variation. In addition, traditional herbal formulae do not have intellectual property that hinders the possible development of herbal extract in developing as a treatment for anti-depression. Thus, KXS_2012_ was developed here by optimizing the pairing of GR-PR and ATR-PO on cell study^14^, and we investigated the functions of KXS_2012_ against depression *in vivo* and *in vitro*.

The CMS rat model is probably the most valid animal model of depression, which aims to model a chronic depression-like state that develops gradually over time in response to stress with more natural induction^15^,^16^. Based on our findings, KXS_2012_ could significantly alleviate depression-like symptoms in CMS-induced depressive rat model. Depression is closely related to decreased levels of neurotransmitters in the brain^30^,^31^. The treatment of KXS_2012_ could restore the low levels of neurotransmitters, i.e. norepinephrine, serotonin, dopamine and 5-HIAA/5-HT, in cerebral cortex, hippocampus and striatum of CMS-induced depressive rats. Abundant evidence indicates that neurotrophic factors play important roles against depression^32^,^33^. According to the results, the most striking feature was that the expressions of neurotrophic factors as well as their receptors could be up-regulated in KXS_2012_-adminstrated group, as compared to CMS depressive group. Our findings implied that KXS_2012_ attenuated depression through up-regulating the levels of neurotransmitters and neurotrophic factors.

The neurogenesis hypothesis suggests that depression is largely caused by an impairment of nerve growth in the brain, which also helps to explain that anti-depressants may take several weeks for restoring neurogenesis to exert an effect^17^,^18^. Here, the potential drug targets and global cellular mechanism on neurogenesis could be addressed through cell study on primary cultured neurons and astrocytes. Our results indicated that KXS_2012_ increased the expression of synaptic vesicle protein, synaptotagmin, at pre-synaptic neurons, and induced the dendritic spine density at post-synaptic neurons. In this regard, KXS_2012_ may serve to promote the possible synapse development between neurons. The development of synapse is also dependent on neurotrophic factors, e.g. BDNF^34^,^35^. In line to this notion, KXS_2012_ increased the expressions of neurotrophic factors in astrocytes through up-regulating cAMP-PKA signaling pathway. Erk1/2, a downstream effector of PKA, was also shown to be participated in the KXS_2012_-induced gene expressions. In addition, the condition medium derived from KXS_2012_-treated astrocytes did not significantly increase the expressions of synaptotagmin and PSD-95 in cultured neurons, suggesting that the potential functional relationship of KXS_2012_ between neurons and astrocytes might be complicated and need further study.

Oxidative stress is considered as a common mechanism associated with most neurodegenerative diseases, e.g. depression. H_2_O_2_ is a potent reactive oxygen species^28^. In H_2_O_2_-stressed astrocyte model, application of KXS_2012_ relieved the H_2_O_2_-induced neurotrophic factor loss and restored the stress-caused phosphorylations of CREB and Erk1/2, suggesting that KXS_2012_ might restore the levels of neurotrophic factors in stressed astrocytes, as to support neurophysiological functions, e.g. neurogenesis. These protective effects were illustrated both *in vitro* and *in vivo* here. Interestingly, Erk1/2 and CREB can be activated not only in response to the pro-growth and pro-survival stimuli, but in response to oxidative stress in neurons. The sustained phosphorylations of Erk1/2 and CREB correlate well with cell death and apoptotic protein expression, which subsequently down-regulate the expressions of neurotrophic factors. Our results implied that KXS_2012_ could down-regulate the phosphorylations of Erk1/2 and CREB in stressed astrocytes, as to improve neuron adaptation^29^.

Considering the major ingredients in KXS_2012_ herbal extract should be crucial for anti-depression, different chemicals within the herbs are known for specific functions. Ginsenosides derived from GR were the main chemicals with strong neurotrophic and neuroprotective effects *in vitro* and *vivo*^36^,^37^,^38^,^39^. 3,6′-Disinapoyl sucrose derived from PR was reported to show obvious anti-depressive effect on depressive animal models, and the efficiency was mediated by increasing BDNF levels and CREB phosphorylation via the CaMKII and ERK1/2 pathway^40^,^41^,^42^. In parallel, ATR-derived α- and β-asarones were shown to reverse learning and memory abilities in dementia mice^43^. Pachymic acid of PO regulated the expression of 5-HT3A receptors in *Xenopus* oocytes^44^. Among all the active ingredients, asarones were considered as toxic. β-Asarone shows genotoxic, hepatocarcinogens and carcinogenicity in rodents. In our previous study, the making up of KXS_2012_ should decrease the toxicity by conversion of β-asarone to a less toxic form α-asarone, as to enhance therapeutic efficacy^14^. Meanwhile, due to compatibility of paired-herbs in TCM that the combination of PO and PR with ATR might help reduce side or adverse effects. Therefore, it is reasonable that KXS_2012_ could be used as a new regimen for anti-depression for the new make-up could exert robust effect against depression through multiple targets, whereas the combination could eliminate toxicity and side/adverse effects.

## Materials and Methods

### Preparation of herbal extracts

The plant materials from Qinping Market in Guangzhou China were morphologically authenticated by one of the authors, Dr. Tina T. X. Dong. The corresponding voucher specimens were deposited in Center for Chinese Medicine of The Hong Kong University of Science and Technology. The herbs were tested to be qualified according to the requirements of Chinese Pharmacopeia (2015 Edition) and Hong Kong Materia Medica Standards. GR-PR was boiled together in a weight ratio of 1:1, and ATR-PO was in 1:2, under moderate heating in 8 volumes of water. In preparing the herbal extracts, 20 g of herb mixture was boiled in 160 mL of water for 2 h and extracted twice. The extracts were filtered and combined, dried under vacuum and stored at −80 °C. KXS_2012_ was obtained by mixing the water extracts of GR-PR and ATR-PO together in 1:5 weight ratio.

The quality control of herbal extracts was described in previous study^7^. An Agilent HPLC coupled with a diode array detector (DAD) system was performed for quality control of the herbal extracts. The samples were analyzed on an Agilent DAD detector G1315C with wavelength of 258 nm and 330 nm. An Agilent zorbax XDB C_18_ column (4.6 mm × 50 mm, 1.8 μm) maintained at ambient room temperature was selected. The mobile phase condition was recorded as follows: 0.1% formic acid in acetonitrile (A) and 0.1% formic acid in water (B). The flow rate was 0.4 mL/min with injection volume of 5 μL. 0–14 min, linear gradient 20.0–42.0% (A); 14–17 min, linear gradient 42.0–75.0% (A); 17–18 min, isocratic gradient 75.0% (A); 18–25 min, linear gradient 75.0–85.0% (A). A pre-equilibration period of 5 min was used between each sample. 3,6′-disinapoyl sucrose, α-asarone and β-asarone were identified by comparison of their retention time (Rt) with those of known standards from the chromatograms. The effluent was further analyzed by an Agilent QQQ-MS/MS system (Agilent QQQ-MS/MS 6410A with an ESI ion source in negative mode). The drying gas temperature was 325 °C; drying gas flow: 10 L/min; nebulizer pressure: 35 psig; capillary voltage: 4000 V; delta electro multiplier voltage: 400 V. The determinations of ginsenoside Rb_1_, Rd, Re, Rg_1_ and pachymic acid were conducted, where astragaloside IV was taken as an internal control. The mass spectra properties of the chemicals were listed in Table S2. Agilent Mass Hunter workstation software version B.01.00 was used for data acquisition and processing. All values are in Mean ±SD, *n* = 3. The established chemical parameters served as the control for repeatability of biochemical analyses.

### Animal experiments

Male Sprague-Dawley (SD) rats (150–180 g, 7-week-old) were obtained from Shanghai SipprBK Laboratory Animals Ltd (Shanghai, China). Animals were hosted on a 12 h light/dark cycle (lights on at 6:00 a.m. and off at 6:00 p.m.) under controlled temperature (22 ± 2 °C) and humidity (50 ± 10%), with standard diet and water *ad libitum*. They were feed for 7 days before use to adapt to the new environment. The experimental procedures had been approved by The Animal Experimentation Ethics Committee of China Pharmaceutical University (No. 2015–0254 in 01/03/2015 for Animal Ethics Approval) and under the guidelines of “Principles of Laboratory Animal Care” (NIH publication No. 80–23, revised 1996). All the efforts were to reduce the suffering of animals. The behavioral tests were conducted in quiet room with soft light and all references stayed the same location.

The procedures of CMS were conducted with some adjustments^11^,^45^. Briefly, a series of stressors were applied onto the animals: (1) water deprivation for 24 h, (2) stroboscopic illumination for 10 h, (3) cage tilt (45°) for 7 h, (4) noise for 10 h, (5) soiled cage (200 mL water in 100 g sawdust bedding) for 12 h, (6) exposure to an empty bottle for 1 h, (7) forced swimming at 8 °C for 6 min, (8) tail-clipping restraint for 6 min and (9) food deprivation for 21 h. These stressors were randomly arranged in one-week and repeated for 6 weeks. The rats in control group were left undisturbed in their home cages except for house maintaining such as cage cleaning. At the end of CMS procedures, sucrose preference test was determined to evaluate the CMS model.

The SD rats were randomly divided into six groups (*n* = 12). The control animals and CMS model were given with saline. For the other four groups, KXS_2012_ at low dose (60.9 mg/kg/day), medium dose (182.7 mg/kg/day), high dose (548.1 mg/kg/day) and imipramine (20 mg/kg/day) were intra-gastrically given 30 min before stress exposure for 6 weeks. Sucrose preference test was carried out at the end of CMS procedures^11^. In Brief, rats in each group were learned to adapt to 2 bottles of 1% sucrose solution (w/v) 72 h before the test, and 24 h later, one bottle of 1% sucrose solution (w/v) was replaced with tap water for 24 h. Then, rats were deprived of water and food for 24 h. Sucrose preference test was conducted at 17:00 p.m., where rats were kept in individual cages with two bottles, one with 100 mL of 1% sucrose solution (w/v) and the other with 100 mL of water. After 3 h, the volumes of consumed sucrose solution and water were recorded and the sucrose preference was calculated by the following formula: sucrose preference = sucrose consumption/(water consumption + sucrose consumption)×100%^45^.

Open field test was carried out at the end of herbal treatment. Briefly, the open field apparatus consisted of a square arena (100 cm × 100 cm × 50 cm). The floor was divided equally into 25 squares. In the test, a single rat was placed in the center of the arena and allowed to explore freely. The time that rats spent into the central area was recorded during a test period of 5 min. This apparatus was cleaned with a detergent and dried after occupancy by each rat. The open field test was started at 9: 00 a.m. in a quiet room^45^.

Forced swimming test was carried out at the end of herbal treatment. Rats in each group were placed in large glass cylinders (50 cm height and 20 cm diameter) with 30 cm height water at 22 ± 2 °C, so that rats were not able to support themselves by hind limbs. The test consisted of two parts: the first 15 min was for pre-swimming and then 24 h later, the swimming behavior was observed in 5 min, and the latency to float was measured and analyzed^46^.

### Measurement of neurotransmitters

The brain tissues were dissected from the manipulated rats. In brief, the rats were sacrificed by decapitation, and the cortex, hippocampus and striatum were separated, respectively, rapidly frozen in liquid nitrogen and kept in −80 °C for storage. The procedure was described: the tissue samples (1 g in 5 mL) were treated by tissue lysate (0.6 mol/L perchloric acid, 0.5 mM Na_2_EDTA and 0.1 g/L L-cysteine), and centrifuged (14,000 g, 15 min, 4 °C) twice. The supernatant was collected and treated with perchloric acid precipitation agent (0.6 mol/L perchloric acid, 1.2 M K_2_HPO_4_ and 2 mM Na_2_EDTA). After centrifugation (14,000 g, 15 min, 4 °C) and filtration, the samples were challenged by HPLC-fluorescence detector (HPLC-FLD, Shimadzu, Japan)^47^.

The chromatographic separation for monoamine neurotransmitters was performed on a Shimadzu HPLC-RF series which was equipped with a degasser, a binary pump, an auto-sampler and a thermo-stated column compartment. The tissue samples were separated on an Agilent zorbax SB-C_18_ (150 mm × 4.6 mm, 5 μm) column. The mobile phase composed of citric acid-sodium acetate buffer (50 mM citric acid, 50 mM sodium acetate, 0.5 mM 1-heptane sulfonate, 5 mM triethylamine, 0.5 mM Na_2_EDTA) (A)-methanol (B) (95: 5, v/v) (pH 3.8); flow rate of 1.0 mL/min; injection volume of 5 μL; emission wavelength of 330 nm, excitation wavelength of 280 nm^19^,^20^,^48^.

### Measurement of neurotrophic factors

The levels of neurotrophic factors were determined by commercial ELISA kits (Abfrontier, Korea) according to the manufacturer’s instructions. The samples were added onto a 96-well plate with coating of anti-rat NGF/BDNF/GDNF antibody and incubated at 37 °C for 90 min. Then, the samples were replaced by biotinylated anti-rat NGF/BDNF/GDNF antibody and incubated at 37 °C for 60 min. After washing four times with PBS, avidin-biotin-peroxidase complex solution was added and incubated at 37 °C for 90 min, which was replaced by tetra-methylbenzidine solution with another incubation of 30 min at 37 °C. At last, 1 M sulfuric acid was added to stop the reaction and absorbance of 450 nm was measured immediately. The absorbance of non-specific binding was taken consideration for sample analysis and each sample in duplicate was employed to minimize inter-assay variation^49^.

### Real-time quantitative PCR

Total RNA was isolated from cell cultures by RNAzol RT reagent (Molecular Research Center, Cincinnati, OH) according to the manufacture’s instruction^12^,^14^. The amounts of RNAs were detected by UV absorbance at 260 nm. The total RNA was used to perform the reverse transcription with Moloney Murine Leukemia Virus (MMLV) reverse transcriptase (Life Technologies, Grand Island, NY), according to the protocol provided by the manufacturer. Real-time PCR was performed using FastStart SYBR Green Master (Roche, Indianapolis, IN). The SYBR green signal was detected by Mx3000P^TM^ muitiplex quantitative PCR machine (BD Biosciences Clontech, San Jose, CA). Primers used were: NGF-S: CAC TCT GAG GTG CAT AGC GTA ATG TC; NGF-AS: CTG TGA GTC CTG TTG AAG GAG ATT GTA C; BDNF-S: GAG CTG AGC GTG TGT GAC AGT ATT AG; BDNF-AS: ATT GGG TAG TTC GGC ATT GCG AGT TC; GDNF-S: GCG CTG ACC AGT GAC TCC AAT ATG; GDNF-AS: CGC TTC ACA GGA ACC GCT ACA ATA TC; TrkA-S: ACC TCA ACC GTT TCC TCC GGT C; TrkA-AS: CTC GAT CGC CTC AGT GTT GGA GA; TrkB-S: CGG GAG CAT CTC TCG GTC TAT G; TrkB-AS: CAA ATG TGT CCG GCT TGA GCT GG; TrkC-S: CAC TGT CTA CTA CCC TCC ACG TG; TrkC-AS: CTC TCT GGA AAG GGC TCC TTA AGG; Synaptotagmin-S: GCT GGG TGA CAT CTG TAC CTC C; Synaptotagmin-AS: CAC CTG GAC TTT CTG GAT CTG CTC; PSD-95-S: TGG TGA CGA AGA GTG GTG GCA AG; PSD-95-AS: CAA AGT GGT AAT CCC GGC CGT C; 18S-S: TGT GAT GCC CTT AGA TGT CC; 18S-AS: GAT AGT CAA GTT CGA CCG TC.

### Cell culture

Cortical and hippocampal neurons, from SD rat embryos at day 18, were isolated and cultured as described previously^50^ with modifications. Briefly, the whole brain was dissected in Hank’s Balanced Salt Solution (Sigma, St. Louis, MO) supplemented with 1 mM sodium pyruvate (Life Technologies) and 10 mM HEPES (Sigma, pH 7.4) without Ca^2+^ and Mg^2+^ to obtain cortex. The tissues were treated with trypsin (Life Technologies, 2.5%) for 20 min, followed by centrifugation at 1,500x g for 4 min or washing with plating medium (DMEM with 10% HS, supplemented with 0.5 mM GlutaMAX, 25 μM monosodium glutamic acid, 100 U/mL penicillin and 100 μg/mL streptomycin) to remove trypsin. Then, the tissues were triturated for several times to get single cells. The number of cells were counted using trypan blue (Life Technologies), and the cells were seeded on a poly-L-lysine (Sigma, 100 μg/mL) coated 60-mm culture plates (10^6^ cells/plate) and incubated at 37 °C in 5% CO_2_. Plating medium was changed to culturing medium (Neurobasal supplemented with 1 × B27, Life Technologies) in 24 h after plating. Cytosine arabinoside (Ara-C, Sigma, 2.5 μM), a mitotic inhibitor was applied onto the cultures on the third day *in vitro* (DIV) to eliminate glial cells. One third of the medium was replaced by fresh culture medium every 5 day afterwards. At DIV 5 or 15, the medium was changed by neurobasal supplemented with 0.1× B27 for 3 h before herbal treatment. The time of herbal treatment was 96 h. Forskolin (Sigma, 50 nM) and BDNF (Alomone lab, Israel, 15 ng/mL) were used as positive controls, respectively^51^.

Astrocytes, from postnatal SD rat at day 1, were isolated and cultured as described previously^52^. The cortex was dissected in Hank’s Balanced Salt Solution without Ca^2+^ and Mg^2+^. After being trypsinized for 20 min, the cortex was washed with culture medium and triturated several times. The culture medium was DMEM supplemented with 10% FBS, 100 units/mL penicillin and 100 μg/mL streptomycin. The cells were centrifuged at 1,000× g for 4 min. The cell pellet was re-suspended in the culture medium. The cells were seeded on plastic culture plates with the density of 2 × 10^4^ cell/cm^2^ and incubated at 37 °C in 5% CO_2_ for 48 h before medium change. The culture medium was changed twice a week. Every time, the astrocytes were pipetted up and down several times to get rid of any loosely adherent oligodendrocytes, microglia and neurons^53^. In herbal treatment, drugs were diluted by DMEM with 0.5% FBS and 100 units/mL penicillin and 100 μg/mL streptomycin, and the medium was changed by fresh DMEM with 0.5% FBS and 100 units/mL penicillin and 100 μg/mL streptomycin 3 h before herbal treatment. The time of herbal treatment was 48 h. H89 (Sigma, 5 μM) and U0126 (Sigma, 20 μM) were applied onto cultures 3 h before herbal treatment, respectively. Forskolin (Sigma, 50 nM) and tetra-decanoylphorbol acetate (TPA, Sigma, 50 nM) were used as positive controls, respectively. In H_2_O_2_-stressed astrocytes, the cells were pre-treated with herbal extracts for 24 h before exposure to H_2_O_2_ (400 μM) for another 24 h. N-acetyl-L-cysteine (NAC, Sigma, 1 mM) was used as a positive control.

### Cell viability assay

Cell viability was assessed by MTT [3-(4,5-dimethyl-2-thiazolyl)-2, 5-diphenyl-2 H-tetrazolium bromide] assay. Cells were plated in 96-well plate for 24 h and treated with drugs for 48 h before adding MTT. In H_2_O_2_-stressed astrocytes, cells were pre-treated with herbal extracts for 24 h before exposure to H_2_O_2_ (400 μM) for another 24 h. NAC (Sigma, 1 mM) were used as a positive control. Then the cells were incubated with MTT for another 3 h at 37 °C. After that, absorbance of 570 nm was measured in a microplate reader (Thermo Scientific, Fremont, CA).

### Protein phosphorylation

Primary cultures of astrocytes were seeded onto 12-well plate to reach the confluence of above 90%, the culture medium was changed to DMEM without serum for 5 h. The inhibitors were added 3 h before herbal treatment. The cells were treated with herbal extracts at different time points (0 min, 5 min, 10 min and 30 min). In stressed astrocyte culture, herbal extracts were applied onto the cells for 24 h, followed by H_2_O_2_ (400 μM) at different time points (0 min, 5 min, 10 min and 30 min). Then, the medium was expelled, and the cells were digested with 2x direct lysis buffer. The degrees of phosphorylation were determined by their specific anti-phospho-kinase and total kinase antibodies. Results are presented as intensities of phospho-bands relative to total bands and expressed as x Basal^54^.

### SDS-PAGE and immunoblotting

The cell lysate was collected, and protein content was determined by Bradford method. Proteins (~20 μg) were separated on 8% SDS-polyacrylamide gels and transferred to a nitrocellulose. Transfer and equal loading of the samples was confirmed by staining the ponceau-S. The nitrocellulose was blocked with 5% fat-free milk in Tris-buffer saline/0.1% Tween 20 (TBS-T), and then incubated in the primary antibodies diluted in 2.5% fat-free milk in TBS-T over night at 4 °C. The primary antibodies were: anti-synaptotagmin (Gift from Prof. H. B. Peng, HKUST), anti-PSD-95 antibody (BD Biosciences), anti-phospho-Erk1/2 (Cell Signaling, Banvers, MA), anti-total Erk1/2 (Cell Signaling), anti-phospho-CREB (Cell Signaling) and anti-total CREB (Cell Signaling). After that, the nitrocellulose was rinsed with TBS-T and incubated for 2 h at room temperature in peroxidase (HRP)-conjugated anti-mouse secondary antibody, or peroxidase (HRP)-conjugated anti-rabbit secondary antibody (Life Technologies), diluted in 2.5% fat-free milk in TBS-T. After intensive washing with TBS-T, the immune complexes were visualized using the enhanced chemiluminescence (ECL) method (GE Healthcare, Piscataway, NJ). The intensities of bands in control and samples, run on the same gel and under strictly standardized ECL conditions, were compared on an image analyzer, using a calibration plot constructed from a parallel gel with serial dilutions of one of the sample^49^,^54^.

### Spine density analysis

Rat hippocampal neurons were seeded on 18-mm coverslips (1.5 × 10^5^ cells per coverslip) coated with poly-D-lysine (1 mg/mL). At DIV 19, hippocampal neurons were changed by neurobasal medium supplemented with 1× B27 1 h before transfecting with pEGFP-N1 (Clontech, Mountain View, CA) using calcium phosphate precipitation, as previously described^55^. For imaging of dendritic spines in EGFP-transfected hippocampal neurons, the images were acquired by Zeiss LSM710 confocal microscope with a 40X oil-immersion objective using z serial scanning mode, and image analyses were performed with Imaris software. For quantification, two to three dendrite segments from each neuron were analyzed. The basal threshold values for the background of all images in each experiment were measured. The average of these threshold values was then applied to all images in the same experiment^56^.

### Statistical analysis

All data were analyzed using one-way ANOVA followed by the Students t-test. Statistically significance were classed as * where P < 0.05; ** where P < 0.01; *** where P < 0.001.

## Additional Information

**How to cite this article**: Yan, L. *et al*. A Chinese herbal decoction, reformulated from Kai-Xin-San, relieves the depression-like symptoms in stressed rats and induces neurogenesis in cultured neurons. *Sci. Rep*. **6**, 30014; doi: 10.1038/srep30014 (2016).

## Supplementary Material

Supplementary Information
