# Supplementary material for: A Chinese herbal decoction, reformulated from Kai-Xin-San, relieves the depression-like symptoms in stressed rats and induces neurogenesis in cultured neurons
Source: Sci Rep. 2016 Jul 22;6:30014. doi: 10.1038/srep30014 (PMC4957105; doi:10.1038/srep30014)
Supplement: Supplementary Information [file srep30014-s1.pdf]

A Chinese herbal decoction, reformulated from Kai-Xin-San, relieves the depression-like symptoms in stressed rats and induces neurogenesis in cultured neurons

Lu Yan[1,2], Qinghua Hu[3], Marvin S. H. Mak[1], Jianshu Lou[1], Sherry L. Xu[1], Cathy W. C. Bi[1,2], Yue Zhu[4], Huaiyou Wang[1,2], Tina T. X. Dong[1,2], Karl W. K. Tsim[1,2,*]

Supporting Information

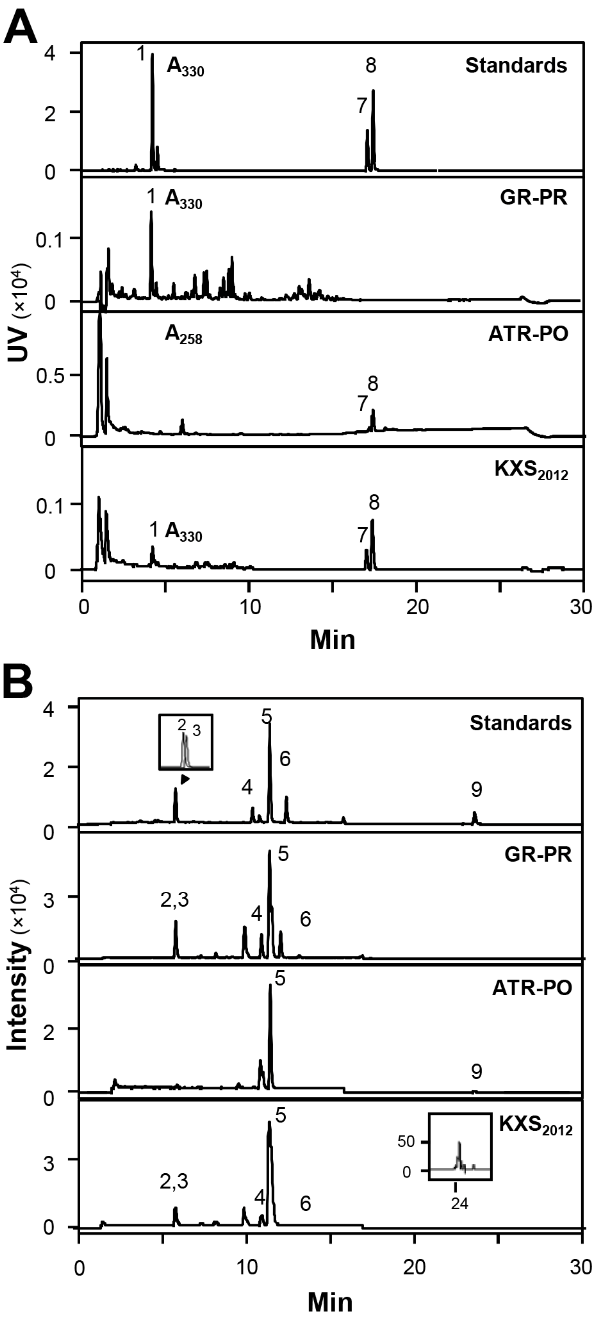

Fig. S1. Standardization of herbal extracts

(A): The standardization of herbal extracts was described in Zhu et al.[7]. The

identification of 3,6'-disnapoyl sucrose (330 nm) (1), α-asarone (258 nm) (8) and β-asarone (258 nm) (7) was made by a HPLC couple with a DAD detector in KXS_{2012} and paired-herb extracts. This HPLC profile served as fingerprint and determination of marker chemicals. **(B):** In chemical assessment, the identification of ginsenoside Rg_1 (2), Re (3), Rb_1 (4), Rd (6), astragaloside IV (5) and pachymic acid (9) was made by a MS detector in standard markers, KXS_{2012} and paired-herb extracts. The detected wavelength was indicated. Representative chromatograms are shown, *n* = 3.

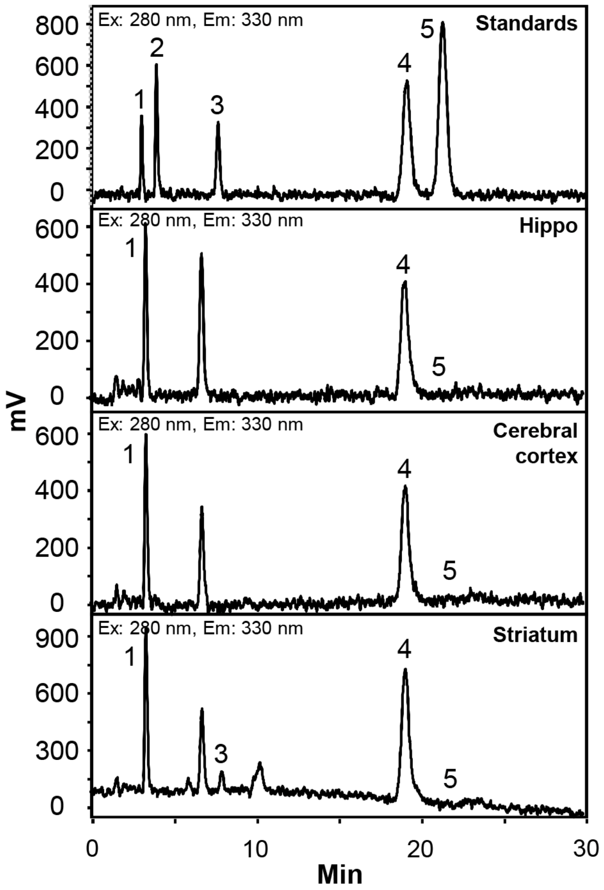

Fig. S2. Chromatograms of neurotransmitters

Different neurotransmitter standards (standard) were prepared in mobile phase. The identification of norepinephrine (1), epinephrine (2), dopamine (3), serotonin (4) and 5-HIAA (5) was made by a fluorescent detector. The extracts of hippocampus (hippo), cerebral cortex and striatum were analyzed. Representative chromatograms are shown, n = 3.

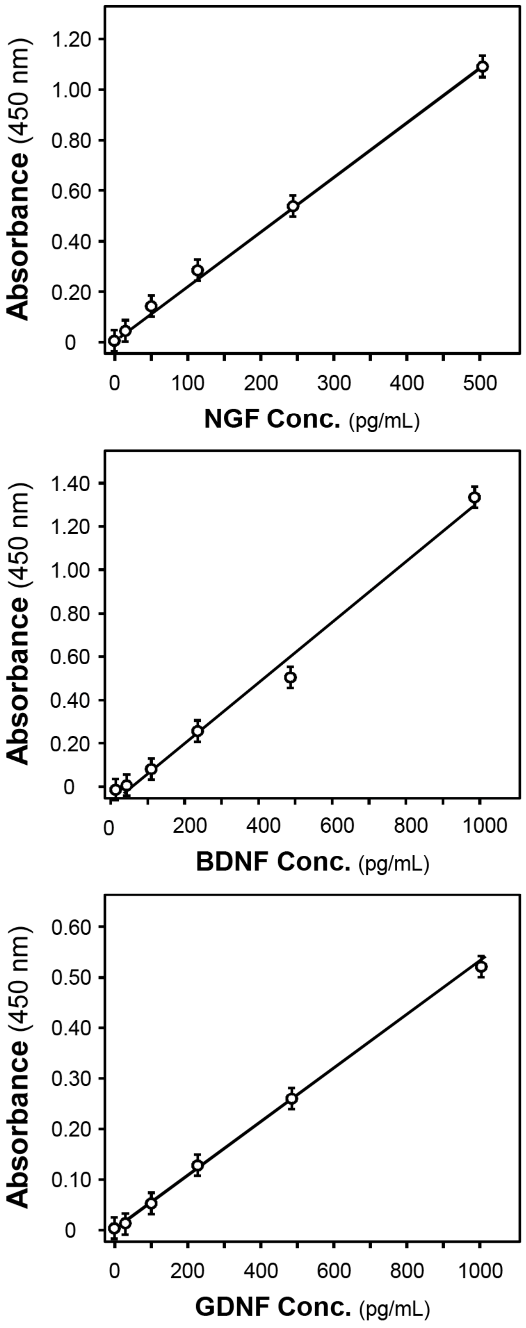

Fig. S3. Standard curves of neurotrophic factors from ELISA

Different concentrations of standard neurotrophic factors (NGF, BDNF and GDNF; 20-1000 pg/mL) were applied onto ELISA kits, and the absorbance was detected

as wavelength of 450 nm. The calibration curves were constructed by plotting the absorbance versus the concentration of each target protein. Each calibration curve was derived from 5 concentrations with duplicate.

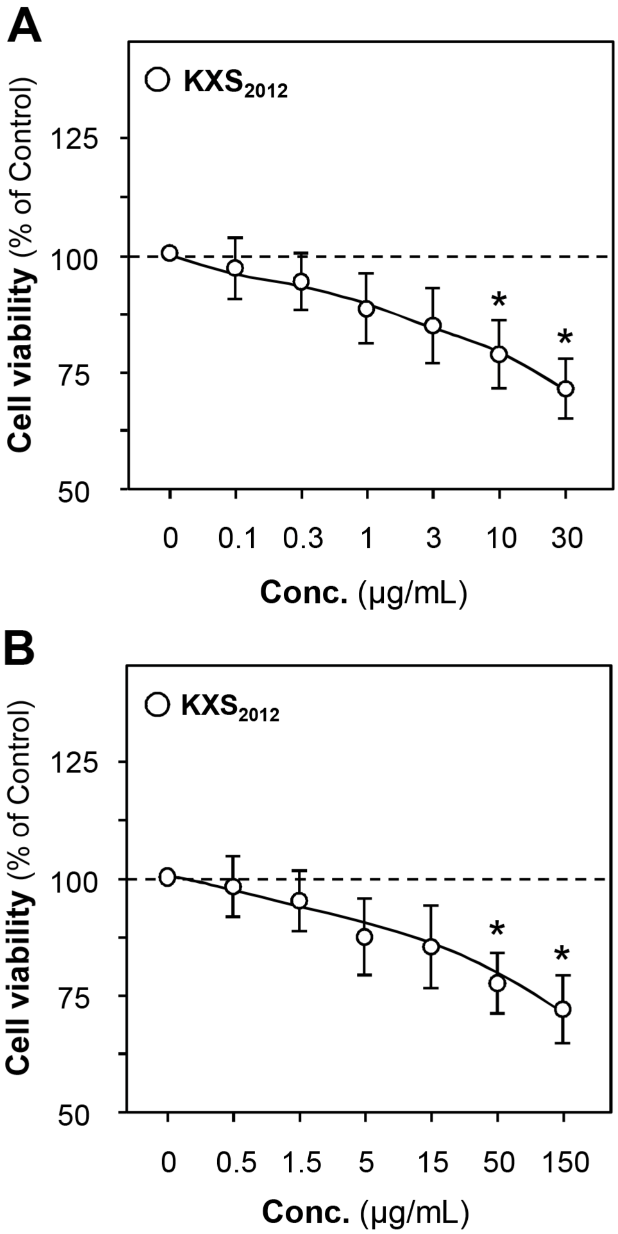

Fig. S4. Cell viability of KXS_{2012} on cultured neurons and astrocytes

Cultured neurons **(A)** were treated with KXS_{2012} (0.1-30 μg/mL) for 96 h, and astrocytes **(B)** were treated with KXS_{2012} (0.5-150 μg/mL) for 48 h. A cell viability and proliferation test (using the colorimetric MTT assay) was performed. Values are in Means ± SEM, each with five samples, n = 5, * p<0.05 compared to the control.

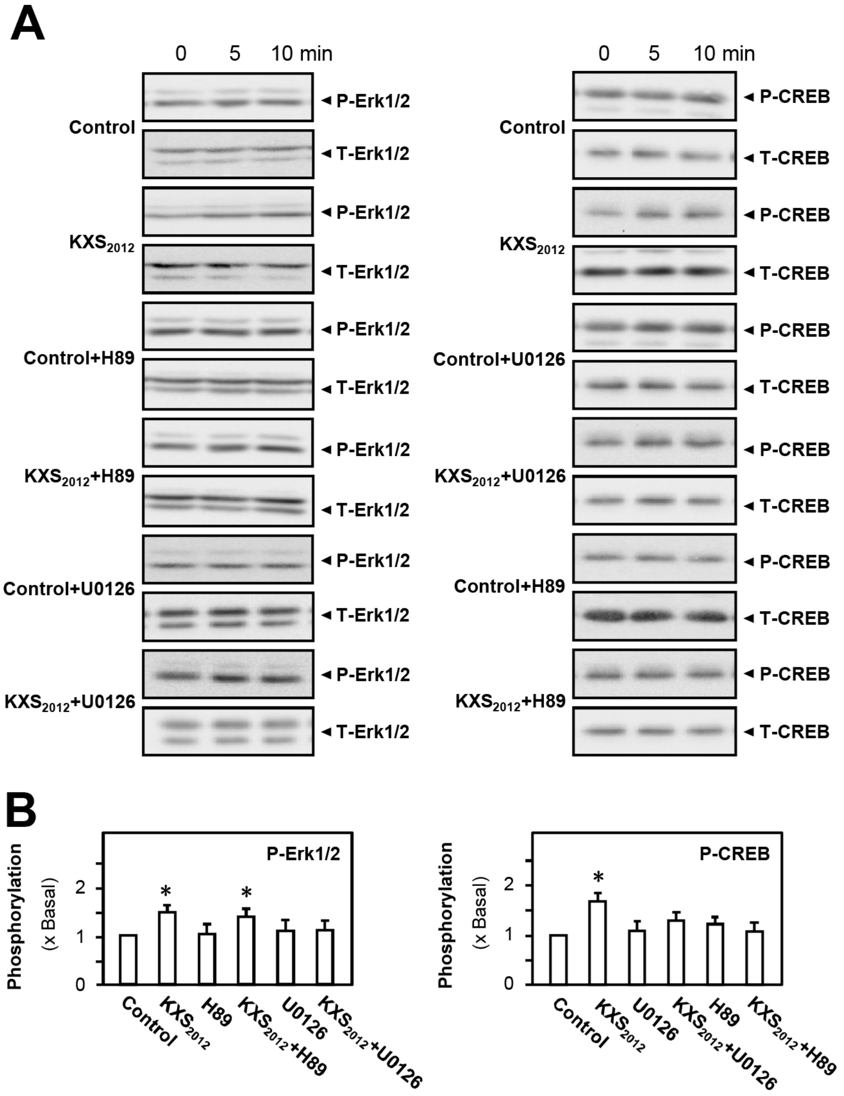

Fig. S5. Crosstalk between cAMP-PKA and MAPK signaling pathway

(A): Cultured astrocytes were serum starved for 5 h. U0126 (20 μM) and H89 (5 μM) were applied onto astrocytes 3 h before KXS_{2012} (15 μg/mL) treatment at different time points. Total Erk1/2, phosphorylated Erk1/2 (both at ~42/44 kDa), total CREB and phosphorylated CREB (at ~40 kDa) were revealed by using specific antibodies. **(B):** Quantification plot of the phosphorylation level at 10 min was shown. Values are expressed as the fold of change as compared to control (x Basal), where control value is set as 1, Mean ± SEM, *n* = 4, * $p<0.05$ compared to the control.

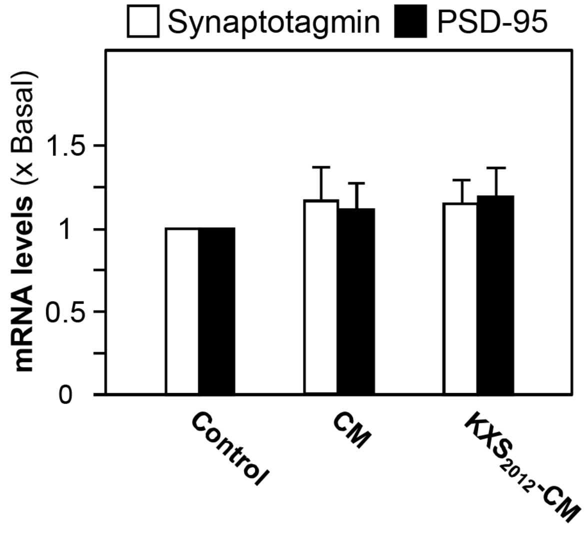

Fig. S6. KXS_{2012} stimulates the expressions of synaptotagmin and PSD-95 in cultured rat cortical neurons

KXS_{2012} (15 μg/mL) were applied onto cultured rat astrocytes for 48 h. Then the cells were washed with 1x PBS and fresh medium was applied onto astrocytes for 24 h. The condition medium was collected and applied onto cultured neurons for

48 h. The mRNA levels of synaptotagmin and PSD-95 were measured by Real-time PCR. Values are expressed x Basal, where control value is set as 1, Mean ± SEM, $n = 4$

Table S1. Calibration of neurotransmitters by HPLC-FLD

Chemical	Equation[a]	Linear range (μg/mL)	Correlation coefficient (R^2)	LOD[b] (μg/mL)	LOQ[c] (μg/mL)
Dopamine	Y=971047X+553123	0.2-10	0.998	0.06	0.2
Norepinephrine	Y=662547X+743936	0.2-10	0.998	0.06	0.2
Serotonin	Y=4000000X+627908	0.2-10	1.000	0.06	0.2
5-HIAA	Y=10000000X+943957	0.1-5	0.997	0.03	0.1

[a] Calibration curves were constructed by plotting the peak area versus the concentration of each analyte. Each calibration curve was derived from six data points (*n* = 6).

[b] LOD referred to the limits of detection in μg/mL of analyte.

[c] LOQ referred to the limits of quantification in μg/mL of analyte.

5-HIAA = 5-Hydroxyindoleacetic acid

Table S2. Mass spectra properties of marker chemicals in herbal extract

Chemical	Formula	Calculated mass	Precursor ion[a]	Fragmentor energy[b]	Collision energy[c]	Product ion[d]
Ginsenoside Rb_1	$C_{54}H_{92}O_{23}$	1108.6	1107.6	250	41	945.5
					49	783.5
Ginsenoside Rd	$C_{48}H_{82}O_{18}$	946.5	945.5	250	33	783.5
					45	621.6
Ginsenoside Re	$C_{48}H_{82}O_{18}$	946.5	945.5	250	41	637.5
					53	475.5
Ginsenoside Rg_1	$C_{42}H_{72}O_{24}$	800.5	799.5	250	21	637.3
					37	475.4
Pachymic acid	$C_{33}H_{52}O_5$	528.3	527.3	250	37	467.2
					41	465.3
Astragaloside IV	$C_{41}H_{68}O_{14}$	784.9	829.5[e]	190	5	829.5
					25	783.2

[a]The detected chemicals had the greatest responses under the negative mode, the [M-H]- was used as the precursor ion.

[b]The fragmentor energy was optimized to have the greatest ionize efficiency.

[c]The collision energy was optimized to have the greatest product ion intensity, which was the key factor in the MRM mode.

[d]Two product ions were used for the MRM analysis. The upper one was used for quantitative analysis and the lower one was for qualitative analysis, which could guarantee the precision of analytes.

[e]The precursor ion of astragaloside IV was [M+HCOOH-H]- under the negative mode.
